# Quantitative Magnetic Resonance Imaging in Perianal Crohn’s Disease at 1.5 and 3.0 T: A Feasibility Study

**DOI:** 10.3390/diagnostics11112135

**Published:** 2021-11-17

**Authors:** Ali Alyami, Caroline L. Hoad, Christopher Tench, Uday Bannur, Christopher Clarke, Khalid Latief, Konstantinos Argyriou, Alan Lobo, Philip Lung, Rachel Baldwin-Cleland, Kapil Sahnan, Ailsa Hart, Jimmy K. Limdi, John Mclaughlin, David Atkinson, Geoffrey J. M. Parker, James P. B. O’Connor, Ross A. Little, Penny A. Gowland, Gordon W. Moran

**Affiliations:** 1 Department of Diagnostic Radiography Technology, College of Applied Medical Sciences, Jazan University, Jazan 45142, Saudi Arabia; aalmansour@jazanu.edu.sa; 2 Translational Medical Sciences Academic Unit, School of Medicine, Faculty of Medicine and Health Sciences, University of Nottingham, Nottingham NG7 2UH, UK; kosnar2@yahoo.gr; 3 National Institute of Health Research Nottingham Biomedical Research Centre at the Nottingham University Hospitals NHS Trust and University of Nottingham, Nottingham NG7 2UH, UK; Caroline.L.Hoad@nottingham.ac.uk (C.L.H.); Christopher.Tench@nottingham.ac.uk (C.T.); Penny.Gowland@nottingham.ac.uk (P.A.G.); 4 Sir Peter Mansfield Imaging Centre, School of Physics and Astronomy, University of Nottingham, Nottingham NG7 2QX, UK; 5 Division of Clinical Neurosciences, Clinical Neurology, University of Nottingham, Queen’s Medical Centre, Nottingham NG7 2UH, UK; 6 Department of Radiology, Queens Medical Centre Campus, Nottingham University Hospitals, Nottingham NG7 2UH, UK; uday.bannur@nuh.nhs.uk (U.B.); christopher.clarke@nuh.nhs.uk (C.C.); khalid.latief@nuh.nhs.uk (K.L.); 7 Department of Gastroenterology, Sheffield Teaching Hospitals NHS Trust, Sheffield S10 2JF, UK; alan.lobo@nhs.net; 8 Department of Radiology, St Mark’s Hospital and Academic Institute, London North West Healthcare NHS Trust, London HA1 3UJ, UK; philliplung@nhs.net (P.L.); r.baldwin@nhs.net (R.B.-C.); 9 Fistula Research Unit, St Mark’s Hospital and Academic Institute, London North West Healthcare NHS Trust, London HA1 3UJ, UK; kapil.sahnan@nhs.net (K.S.); ailsa.hart@nhs.net (A.H.); 10 Department of Gastroenterology, The Pennine Acute Hospitals NHS Trust, Greater Manchester, Crumpsall M8 5RB, UK; jimmy.limdi@nhs.net; 11 Department of Gastroenterology, Salford Royal NHS Foundation Trust, Manchester Academic Health Sciences Centre, Salford M6 8HD, UK; john.mclaughlin@manchester.ac.uk; 12 Centre for Medical Imaging, University College London, London W1W 7TS, UK; d.atkinson@ucl.ac.uk; 13 Centre for Medical Image Computing, Department of Medical Physics and Biomedical Engineering, University College London, London WC1V 6LJ, UK; geoff.parker@ucl.ac.uk; 14 Bioxydyn Limited, Manchester M15 6SZ, UK; 15 Quantitative Biomedical Imaging Laboratory, Division of Cancer Science, University of Manchester, Manchester M13 9PL, UKross.little@manchester.ac.uk (R.A.L.)

**Keywords:** perianal Crohn’s disease, quantitative MRI, MT, DWI, T2, DCE

## Abstract

Perianal Crohn’s Disease (pCD) is a common manifestation of Crohn’s Disease. Absence of reliable disease measures makes disease monitoring unreliable. Qualitative MRI has been increasingly used for diagnosing and monitoring pCD and has shown potential for assessing response to treatment. Quantitative MRI sequences, such as diffusion-weighted imaging (DWI), dynamic contrast enhancement (DCE) and magnetisation transfer (MT), along with T2 relaxometry, offer opportunities to improve diagnostic capability. Quantitative MRI sequences (DWI, DCE, MT and T2) were used in a cohort of 25 pCD patients before and 12 weeks after biological therapy at two different field strengths (1.5 and 3 T). Disease activity was measured with the Perianal Crohn’s Disease Activity index (PDAI) and serum C-reactive protein (CRP). Diseased tissue areas on MRI were defined by a radiologist. A baseline model to predict outcome at 12 weeks was developed. No differences were seen in the quantitative MR measured in the diseased tissue regions from baseline to 12 weeks; however, PDAI and CRP decreased. Baseline PDAI, CRP, T2 relaxometry and surgical history were found to have a moderate ability to predict response after 12 weeks of biological treatment. Validation in larger cohorts with MRI and clinical measures are needed in order to further develop the model.

## 1. Introduction

Perianal Crohn’s disease (pCD) is a common and often debilitating manifestation found in a third of Crohn’s disease (CD) patients [1], causing various symptoms, such as perianal discharge, pain, abscess formation and bleeding, that negatively affect a patient’s quality of life. Patients may require multiple medical and surgical interventions and it represents a major therapeutic challenge. To date, a number of clinical variables have been associated with disease outcomes in pCD. Colonic [2] or rectal disease location [3,4], fistula complexity [5,6], female gender [6], presence of a rectovaginal fistula [6] and absence of a stoma [7] or a surgical history [5] are independently associated with adverse outcomes.

Due to the absence of reliable disease activity measures, it is very difficult to quantify inflammation and optimise therapy in patients with pCD. Clinical measurements such as the Perineal Disease Activity Index (PDAI) [8] and Fistula Drainage Assessment [2] are subjective and prone to inter-operator variability, making these indices less sensitive to assess responsiveness to medical therapy. This absence of useful metrics limits assessment of individual patients within day-to-day healthcare and also limits assessment within clinical trials of therapeutic agents [9].

Magnetic Resonance Imaging (MRI) is a non-invasive approach and allows for morphological evaluation and perianal fistula classification [10]. Pelvic MRI has been shown to be useful in defining the anatomy of the pelvic region in pCD and in measuring the inflammatory activity of the fistula [11,12]. MRI is the preferred test for assessing pCD and its response to biological therapy [13,14,15,16]. However, radiological scoring of features in conventional MRI sequences are prone to inter-observer variability. The Van Assche score was originally devised as a subjective MRI index for pCD [17] and provides combined information of anatomical fistula description and features reflecting inflammatory activity. Evaluation of this score is difficult because no gold standard exists in determining fistula healing. Recent modifications of this score have been put forwarded but have not yet been externally validated [18,19].

Quantitative MRI sequences, such as diffusion-weighted imaging (DWI), dynamic contrast enhancement (DCE), magnetisation transfer (MT) and T2 relaxometry, offer opportunities to improve diagnostic capability. Both DCE and DWI have shown potential in the assessment of activity in pCD [13,20], while MT may be a feasible tool in distinguishing inflammatory from more fibrotic fistulae [21]. Although not applied previously to pCD, T2 relaxometry has been shown to improve the detection of edema in the myocardium [22]. These quantitative MRI measures have never been investigated together prospectively in a patient cohort with active pCD. Moreover, it is not yet clear which platform (1.5 Tesla (T) or 3 T) is optimal for the best sensitivity of these measures whilst minimising artefacts and distortions.

The aims of this exploratory study were:

1. To measure disease activity within a pCD patient cohort using quantitative MRI sequences (DWI, DCE, MT and T2 relaxometry) and clinical parameters before and after biological therapy.
2. To investigate the repeatability of the quantitative data and compare the utility of both 1.5 and 3 T MRI platforms.
3. To design a pCD MRI model to predict response to therapy at baseline.
4. To investigate the inter-relationship between these MRI sequences.

## 2. Materials and Methods

### 2.1. Study Population

This was a multicentre prospective cohort study (3 sites for MRI scanning, 4 sites for recruitment). All participants were about to commence biological treatment under an approved licence as part of their standard clinical care. Participants had to meet the following inclusion criteria: age 16–75 years with active pCD (as defined by clinical assessment and a Perianal Disease Activity Index (PDAI) score of >4), and a clinician decision to start anti-tumour necrosis factor (TNF) or Ustekinumab therapy. Exclusion criteria included absence of a diagnosis of CD, perianal fistulising disease not secondary to CD, malignant disease, significant cardiovascular or respiratory disease and hepatic disease or renal failure, pregnancy or breastfeeding, inability to consent, history of proctectomy and unwillingness to undergo biological therapy or a contraindication to MRI. All participants gave written informed consent, and the study was approved by the National Health Service Ethics Research Committee ([16]/EM/0433).

**Figure 1:**
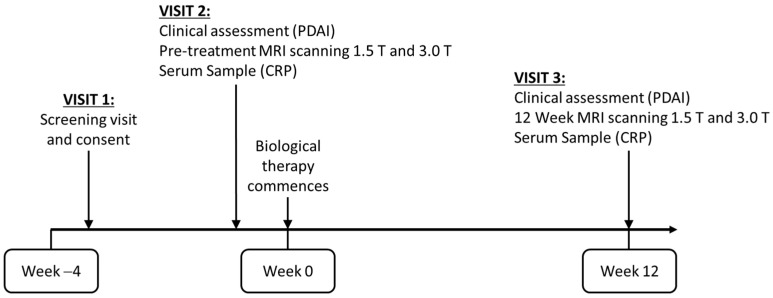
Patient journey. Nottingham and Manchester patients attended a maximum of 3 visits, and London patients a maximum of 5 visits, as the MRIs were not on the same day. Their clinical assessment and serum samples were collected at the 1.5 T visit.

Participants attended a maximum of three research visits (see Figure 1 for patient journey). Up to four weeks before the start of biological therapy, participants attended a screening visit to check eligibility. The second visit involved the first MRI appointment (before starting biological therapy). Visit three was for the second MRI (12 weeks after biological treatment onset). At each MRI scan appointment, participants also underwent a clinical assessment of their pCD according to the Fistula Drainage Assessment and PDAI and serum samples were collected to analyse C-reactive protein (CRP).

### 2.2. MRI Image Acquisition

Participants were scanned using the following 1.5 and 3.0 T scanners using either pelvic or torso phased array coils; Nottingham site—1.5 T GE HDxt Signa (GE Healthcare, Milwaukee, WI, USA) and 3.0 T Philips Ingenia scanners; London site—1.5 T Philips Achieva and 3.0 T Philips Achieva scanners; Manchester site—1.5 T Philips Intera and 3.0 T Philips Achieva scanner (Philips, Best, The Netherlands).

All participants were scanned in a feet-first supine position for all visits. The details of the MRI sequence parameters used at both 1.5 and 3.0 T are given in Appendix A. Brief details are given below.

DWI and MT (on and off) sequences were added to the standard clinical protocol undertaken on the 1.5 T MRI scanner, which were sagittal, coronal and axial T2-weighted fast spin echo (FSE) imaging sequences, and coronal and axial oblique short tau inversion recovery (STIR) sequences. For DWI b-values of 0, 600 s/mm^2^ were acquired on the GE platform and 100, 300, 600 s/mm^2^ were acquired on the Philips platform.

At 3 T, DWI, DCE, T1, T2 and MT sequences were added to the standard clinical protocol, which consisted of coronal, axial and sagittal T2-weighted turbo spin echo, oblique axial and oblique coronal fat-suppressed T2-weighted and pre/post-contrast-enhanced T1-weighted scans. Across all 3.0 T scanners, DWI data were acquired with b-values of 0, 100, 300 and 600 s/mm^2^.

T2 was measured from fast spin-echo sequences in the coronal-oblique plane, acquired at two different echo times (TE) of 80 and 7.30 ms, keeping all other imaging parameters constant. The MT and the T2 sequences were planned parallel to the anal canal to include as much of the fistula region as possible.

The DCE protocol involved 2 stages: firstly, a variable flip angle (FA) (2°, 10°, 20°, 30°) T1 3D gradient-echo sequence without any fat suppression [23] was used to generate a pre-contrast T1 relaxation time map of the tissue; secondly, dynamic imaging was carried out using a 4D THRIVE (T1-weighted High-Resolution Isotropic Volume Examination) sequence with fat suppression, FA = 15°. Imaging was performed over 12 slices per dynamic with a temporal resolution of 5 ms. Gadoteridol (Bracco International B.V, Strawinskylaan, Amsterdam, the Netherlands) was intravenously injected (0.2 mmol/kg) at a rate of 3.0 mL/s using an automated injection pump after 6 pre contrast acquisitions to acquire adequate baseline data. This was followed by a 20 mL 0.9% saline flush administered at the same rate. The delayed reconstruction of this data allowed for immediate acquisition of the T1-post-contrast data.

### 2.3. MRI Image Analysis

Quantitative maps were generated for the DWI, MT, T2 and DCE data on a voxel-by-voxel basis. DWI maps were calculated as an apparent diffusion coefficient (ADC) using a mono-exponential decay from all b-value data at 3 T and only 0 and 600 s/mm^2^ for 1.5 T. MT ratio (MTR) maps were generated from the MT-on and MT-off data [24]. T2 was calculated using a mono-exponential decay from the 2 different echo times of the FSE sequence. The Extended Tofts Model (ETM) [25,26], using a population arterial input function [27], was used to generate the DCE parameters (vascular transfer constant (*K*^trans^), the volume fraction of the extravascular, extracellular space (*v_e_*), and the volume fraction of the plasma space (*v_p_*) from the tissue uptake curves. T1 maps were generated from the pre-contrast variable flip angle gradient echo sequence [23], taking into account the differences in fat saturation between the VFA and DCE sequences (additional details in Appendix B).

#### 2.3.1. Radiological Evaluation

Assessment of all the MRI data was carried out at a single site by one of three specialist consultant gastrointestinal (GI) MRI radiologists (UB, LK and CC) (with >5 years of MRI experience), who were blinded to the clinical results. Both visits of the same patient were assessed by the same radiologist. For each patient, a GI radiologist calculated the Van Assche score [17] using only 1.5 T images before the 3 T images were viewed. This score ranges from 0 to 22, with higher scores indicating more severe disease.

**Figure 2:**
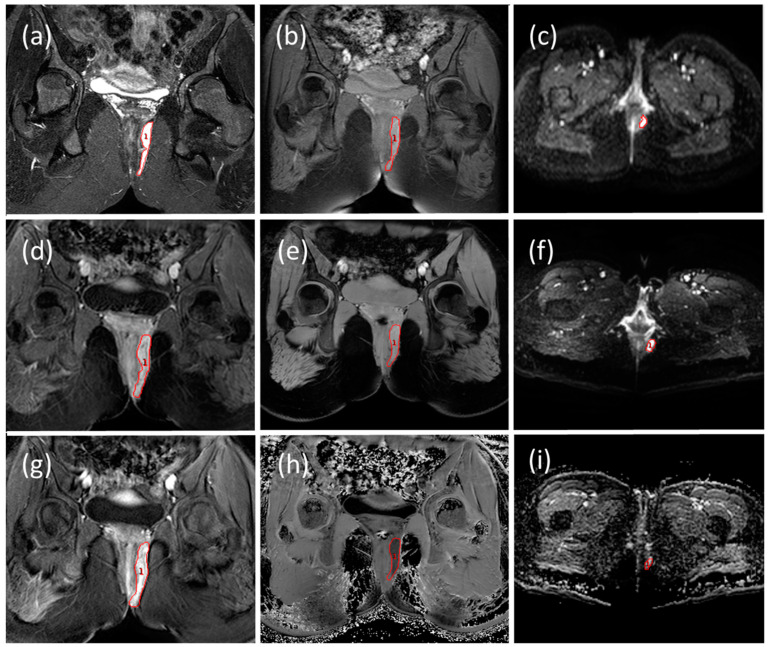
Patient with clearly visible fistula. (**a**) 1.5 T coronal STIR image where a radiologist drew the ROI and (**b**) 1.5 T MT ‘on’ image. (**c**) 1.5 T DWI b0 image shows where the ROI were copied. (**d**) 3.0 T coronal T1 post-contrast image where a radiologist drew the ROI and (**e**) 3.0 T MT ‘on’ image. (**f**) 3.0 T DWI b0 image where the ROI was copied. (**g**) 3.0T DCE image (**h**) 3.0 T MTR MAP and (**i**) 3.0 T ADC MAP with ROI shown from where results were calculated.

The GI radiologist then defined diseased tissue regions of interest (ROIs) on MRI scans on all slices where the fistulae (including all branches) were visible using the coronal FSE STIR at 1.5 T (Figure 2a) and on the T1 post-contrast coronal images at 3 T (Figure 2d).

Once the regions were identified on the coronal STIR and T1 post-contrast sequences, the same regions were manually co-located to the DWI axial, MT coronal, DCE coronal and T2 coronal raw images, (Figure 2) using Analyze^®^ software (Biomedical Imaging Resource, Mayo Foundation, Rochester, MN, USA). These ROIs could then be directly copied on to the calculated MR parameter maps to extract ROI data histograms. These MR parameter histograms showed the data had outliers and skewed distributions and therefore the median value of the ROI was calculated and used as the data value of the specific MR parameter for each ROI drawn.

#### 2.3.2. Repeatability of Quantitative Measurements

**Figure 3:**
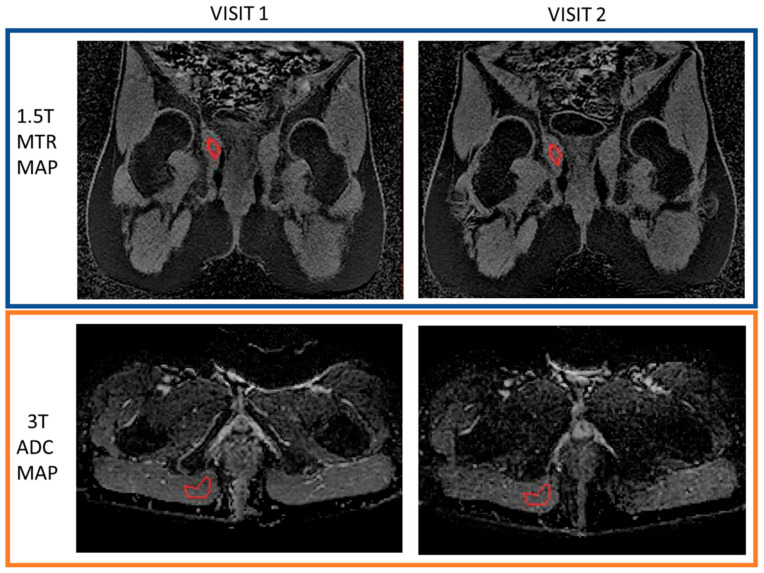
An example of muscle area ROIs drawn on MTR (1.5 T) and ADC (3.0 T) maps for both visits.

To assess the repeatability of these measurements, muscle tissue in the pelvis of the participants was used as a control region. ROIs of approximately 3 mL were drawn by the same observer, in co-located regions of the muscle across visit 1 and visit 2 data for all quantitative imaging sequences (Figure 3), except the DCE data since only the fistula region had been fitted using the Extended Tofts Model. The size of the ROI (3 mL) was towards the lower end of the range of fistula volumes and would therefore represent the worst-case data in terms of the impact of voxel averaging.

### 2.4. Clinical Evaluation

Clinical assessment was performed by local investigators. The PDAI was scored by interviewing the patient and undertaking an examination of the perianal region on the same day as the MRI scan. The PDAI includes five items: degree of induration, type of perianal disease, restriction of sexual activity, restriction or pain with activities of daily living and the presence or absence of discharge. Scores ranged from 0 to 20, with higher scores indicating more severe disease [8].

Blood samples for CRP (mg/L) were taken at each MRI visit and assessed at the biochemistry department of Nottingham University Hospitals NHS Trust, St Mark’s Hospital and Academic Institute, Sheffield Teaching Hospitals and Salford Royal NHS Foundation Trust.

### 2.5. Statistical Methods

This was an exploratory analysis with no a priori sample sizes estimated, so no formal testing was undertaken. It was assumed that continuous data were normally distributed and so they were expressed as a mean and standard deviation (SD). Bland–Altman (B-A) plots were drawn to provide limits of agreement (LOA) between data sets for the muscle repeatability data. The Bland–Altman bias (mean difference between Visit 1 and Visit 2) was also calculated; a value close to zero indicates there was no systematic bias between the measurements [28]. Difference in data sets between time points is presented as an estimated difference of mean with a 95% confidence interval (CI). Individual patient differences were also assessed using the definitions of meaningful change for clinical parameters ([29] which is defined as more than 50% change of the standard deviation of the baseline data. For MRI parameters, only differences greater than the B-A LOA of the muscle were defined as a change.

To predict prospectively those subjects likely to respond to treatment, a model of the follow-up PDAI score was built based only on baseline factors. In the absence of an a priori specification, the model was kept simple, with validation left to future studies. A general linear model was constructed using baseline data considered important for response. Only first-order terms were included, with no interactions considered due to limited sample size. The decision to include or exclude factors was not based on statistical significance, but on clinical importance [2,3,4,5,6,7], with apparent impact on the R^2^ value used as a guide. The strength of the relationship between the different MRI parameters, MTR, T2, T1, *K*^trans^, *v_e_*, *v_p_* and ADC, was assessed using Pearson’s correlation coefficients across pooled data from both the baseline and 12-week MRI visits. All analyses were carried out using GraphPad Prism (version. 8.1.2; GraphPad Software, Inc. San Diego, CA, USA).

## 3. Results

### 3.1. Patient Characteristics

Twenty-five participants were recruited. Six participants were withdrawn from the analyses for the following reasons. Two patients were withdrawn prior to the first MRI study day, of which one failed screening and the other started treatment before the MRI. Four patients were withdrawn prior to the second MRI study day, of which two participants stopped treatment prior to the 12-week assessment, one participant was later diagnosed with rectal malignancy, and one patient withdrew from the study. In addition, one participant had extremely poor MRI data quality due to motion artefacts and was withdrawn from data analysis. Where specific sequence data were missing in some participants, these were excluded in individual analyses. The consort diagram and participant demographic characteristics are presented in the supplementary information.

### 3.2. Changes in Clinical and MRI Parameters after Treatment

Only two MRI parameters altered their mean values after 12 weeks of treatment, with a decrease in T1 and an increase in ADC at 1.5 T seen. None of the rest of MRI parameters from the diseased tissue altered their mean values (Table 1). The PDAI score and CRP showed a decrease from baseline to 12 weeks. Individual data for all measures can be found in the Supplementary Information (Figures S2–S4). A graphical representation of the individual changes for each subject are shown in Table 2, showing the variability of response to treatment.

### 3.3. Repeatability of Quantitative Measurements

**Figure 4:**
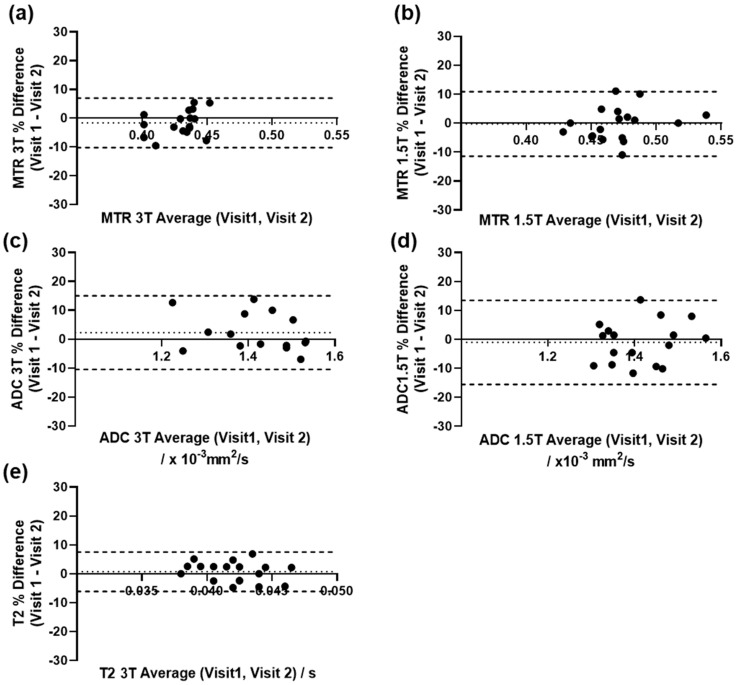
Bland–Altman plots for repeatability muscle data from 3 T (**a**,**c**,**e**) and 1.5 T (**b**,**d**). Data shown as % change from average. Limits of agreement are shown as dashed lines and bias is shown as dotted line.

Bland–Altman plots of the repeatability data are shown in Figure 4 with bias and limits of agreement shown on the graphs. The ADC data at 1.5 T showed the poorest repeatability in the muscle data; the T2 measurements at 3 T showed the best.

### 3.4. A Baseline Model to Predict Response at Week 12

Based on factors reported to be independently associated with adverse outcome, a linear model was constructed to estimate follow-up PDAI score from baseline features. Included factors were disease location (colonic/rectal; *R_inv_*), fistula complexity (*Complex*), sex, presence of a rectovaginal fistula (*RV_fistula_*), stoma and surgical history. Baseline PDAI was also added, as was baseline CRP and baseline T2 (*T*2_1_), as these tended to explain more variance than most of the associated features. The final model specification was:

$$PDAI_{2}=-8.4+0.72\times PDAI_{1}-0.07\times CRP_{1}+0.13\times T2_{1}-1.7’R_{inv}-1.17\times Complex+2.6\times Sex\;+1.7\times RV_{fistula}+4.6\times Stoma-5.1\times Surgery$$

which has an R^2^ of 0.7 for the subjects in this study and where subscript 1 denotes a measurement from the baseline visit and subscript 2 the follow-up at 12 weeks. From these variables, the T2 and the surgical history had the largest statistical effect sizes.

### 3.5. Correlation between MRI Parameters

**Figure 5:**
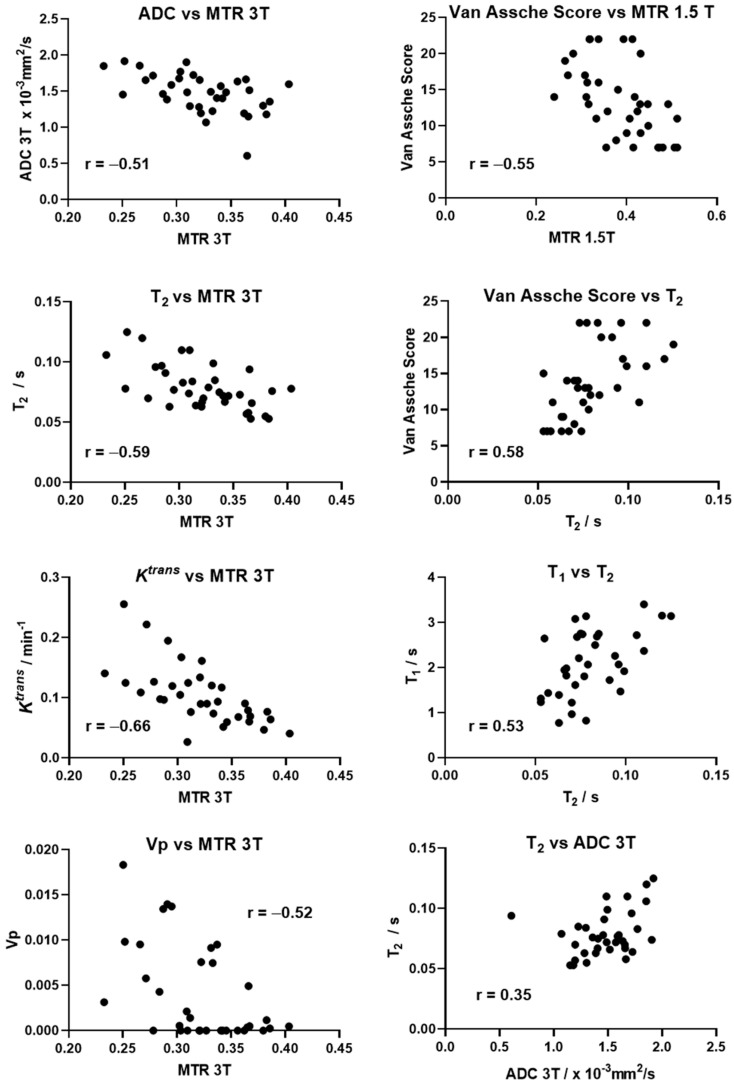
Graphs of correlation between some of the MRI quantitative parameters with the Pearson correlation coefficients shown on the graph.

MTR measured at 3 T negatively correlated with the following parameters: ADC (Pearson correlation coefficient r = −0.51, n = 35), T2 (r = −0.59, n = 36), *K*^trans^ (r = −0.66, n = 34), and Vp (r = −0.52, n = 34). T2 positively correlated with T1 (r = 0.53, n = 34), ADC (r = 0.35, n = 36) and the Van Assche Score (r = 0.58, n = 36). MTR measured at 1.5 T negatively correlated with the Van Assche Score (r = −0.55 n = 36). There was no correlation between any of the other MRI parameters. Figure 5 shows scatter plots for these correlations. All correlation data are presented in the Supplementary Information Table S2.

## 4. Discussion

In this study, we investigated the feasibility of quantitative MRI sequences (MT, DWI, T2, and DCE) for the assessment of perianal fistulae in CD, their utility for the assessment of disease activity and variations associated with treatment. We found that the T2, ADC and MTR parameters were repeatable in the muscle tissue, with the Bland–Altman limits of agreement being relatively low (<20% of median parameter value), the 1.5 T data sets showing poorer repeatability compared to 3 T for both MTR and ADC and the T2 data sets acquired on the 3 T platforms having the smallest limits of agreement. However, the estimated difference in MR values (at 12 weeks treatment when compared to baseline) were all close to 0, except the Van Assche score and T1, which decreased, along with the clinical indicators PDAI and CRP. ADC measured at 1.5 T showed a small increase between the baseline and 12-week measurements. However, a heterogeneous response was seen across subjects adding additional variability into the small sample investigated, with many of the MRI parameters not changing by amounts more than the limits of agreement of the measurements.

Baseline symptoms measured using PDAI and disease activity measured by CRP and T2 were found to have a moderate ability to predict response after 12 weeks of biological treatment, along with other known risk factors, such as sex, surgical history and fistula complexity. This relatively modest predictive value (R^2^ = 0.7) may be attributed to the heterogeneous response to treatment and the early assessment at 12 weeks. Prediction of PDAI may be important for identifying response to treatment, but validation, improvement, and accurate parameter estimation for this model should be investigated in future studies.

We observed negative correlations between MRI measures that have been used previously as markers of inflammation in T2, ADC and *K*^trans^ and those of fibrosis in MTR. Previous studies of luminal Crohn’s disease have shown histological correlation of increased T2 signal related to the presence of edema in the bowel wall [30,31] and lower signal in T2-weighted images and low enhancement on post-contrast T1-weighted images for fibrotic disease [32]. Fibrotic disease has been associated with an increase in MTR due to the relative increase in collagen content [33]; hence, this negative correlation is expected. Dynamic contrast-enhancement is sensitive to tissue inflammation, due to its ability to quantify the functional status of tissue microvasculature, and has been shown to have great potential as a non-invasive measure of pCD activity [16,34,35]. These correlations may suggest that as tissue inflammation recedes with treatment, the residual tissue may have a higher MTR due to the residual fibrosis, or that the MTR signal may also be affected by inflammation. However, in the absence of histopathological correlation, this interpretation is speculative and further work is required to determine these relationships.

Ziech et al. [16] performed the first study using quantitative DCE to evaluate disease activity in patients with pCD. They observed a correlation between the quantitative parameters and PDAI. They found that *K*^trans^ in their responder’s group had decreased considerably 6 weeks after the start of treatment with anti-TNF therapy (*p* = 0.027). However, we were underpowered to split the data by responder status and are therefore unable to confirm this observation. The values we calculated across our cohort were lower than those of Ziech; however, this may have been due to different assumptions in the modelling process, including the definition of Arterial Input Function, and the use of an assumed blood T1 by Ziech Lefrancois et al. [36] also evaluated semi-quantitative DCE parameters in a retrospective study of 43 patients. This study investigated the ability of intravoxel incoherent motion (IVIM)-DWI sequences combined with DCE parameters to differentiate between active and inactive fistulas and found improved diagnostic capability with the inclusion of the DCE parameters compared to IVIM-DWI alone. No direct comparison can be made with this study, as we did not categorise the fistulas as active or inactive and used fully quantitative DCE parameters; however, it highlights the strength of combining MRI quantitative parameters for improved diagnostics.

DWI sequences have recently been studied for the evaluation of fistulae [20,37,38,39]. In a retrospective study involving 24 patients with 41 lesions (23 active lesions and 18 inactive perianal fistulae) [20], the mean value for ADC in the active perianal fistula group (0.908 ± 0.171 × 10^−3^ mm^2^/s) was significantly lower than for the inactive group (1.124 ± 0.244 × 10^−3^ mm^2^/s). A significant decrease in ADC in active pCD was replicated in another study [37]. These ADC results were lower than those measured in our study; however, this could be attributed to the different b-values used in the previous studies (b = 0, 1000 s/mm^2^) and the fact that all inflammatory tissues were included in our study, compared to just fistula tracks in the previous studies. Dohan et al. [38], who used b = 0, 600, 1000 s/mm^2^, measured ADC values in their fistula tracks of 1.41 (1.37–1.53) 10^−3^ mm^2^/s, similar to the results found in this study. Our study was underpowered to allow us to attempt a similar analysis of splitting the cohort by responder status. The use of different b-values across the two field strengths also meant that there were different weightings to the perfusion effects (low b-values) in the measured ADCs.

We did not detect a change in MTR after treatment in this study. Previously, in a group of 29 patients with pCD, Pinson et al. [21] found MTR to be significantly lower in inactive fistulae; however, their study did not look at the effects of treatment. The numerical results between the studies are not directly comparable due to the use of different off-resonance frequency pulses, which can have substantial effects on the MTR measured.

To the best of our knowledge, quantitative T2 relaxometry has not been studied in pCD. STIR or fat-saturated T2W images are most sensitive for detecting fluid and inflammation, including fistula tracts. In this study, we observed no difference in T2 before and after treatment. This might be explained because we defined the fistula in the follow-up visit to include only the visibly ‘inflamed’ regions of the fistula, which could automatically lead to similar T2 measures possibly covering a smaller region. In addition, our small sample size and heterogenous response to treatment may have also influenced the results. Future work should consider combining T2 signal intensity and fistula volume into a composite measure. This progress will be important in providing further data to the debate on whether gadolinium contrast is needed for pCD assessment or whether STIR images have sufficient sensitivity [40]

Individual fistula volumes changed considerably over the 12-week treatment period, with a trend for volume decrease from baseline to 12 weeks in this study. Non-significant changes in volume have been previously found in retrospective analyses of 18 pCD cases using fat-suppressed T2-weighted images [41]. Time for the volume assessment in the study was in the region of 4 min with excellent inter-observer agreement [41].

We found that baseline symptoms, as measured by PDAI, CRP and the baseline T2 value, along with other known risk factors, had a moderate ability to predict response as early as 12 weeks. The quantitative MRI measures together with a clinical core-outcome set could potentially form the basis of objective assessments of future therapies in pCD. This is an encouraging result that warrants further investigation, including revalidation in a larger sample cohort with a longer follow-up.

Our study had a number of limitations. pCD studies have been hampered by a lack of an externally validated gold-standard measure. Our sample size was relatively small, which inherently introduces a level of variability in all readouts. Variability of the MRI parameters from intra- and inter-observer definitions of the fistula ROI were beyond the scope of this study, but will have contributed to variability in the measured parameters and may provide further reasons why no changes were observed, particularly for small fistula volumes, where a small change to the ROI size could have a large effect on the measured parameter. It is currently not possible to match the ROIs from baseline to 12-week scans. Although the limits of agreement for the muscle data were quite low across the different MRI parameters, around 50% of the MT and ADC data from the fistula regions did not show a change greater than these limits. This again could be due to the definitions of the ROIs, which would bias the data to the visible and hence actively diseased tissues, keeping parameters more constant. The definition of the ROI for quantitative studies warrants further investigation.

It is already known that MR-based scoring systems show variability between radiological observers [18]; however, a previous study by Lung et al. [41] showed excellent observer agreement (ICC > 0.9) for the measurement of fistula volume from T2-weighted fat-saturated data, although the range of fistula volumes that these data represented was not published. Additionally, for each case, only two MRI scans were evaluated (baseline and at 12-week follow-up). Twelve weeks may be too early to allow any significant changes in MRI signal to be detected, especially in patients with a heavy disease burden. Future studies should include serial MRI scans for patients using prolonged follow-ups in order to gather evidence on the best timing for MRI scans. Moreover, attempts should be made to use the baseline ROI when measuring changes in signal intensity at follow-up and not rely on visible disease at the time of data acquisition. This will be essential in order to correctly measure the change in disease burden over time.

## 5. Conclusions

Our study provides evidence that (if available) quantitative pCD MRI imaging is optimally performed at 3 T (compared to 1.5 T). Baseline T2 relaxometry, together with CRP and clinical symptoms, shows promise as a predictive tool to identify those that will respond to treatment. However, further work is needed to define the role of these MRI sequences in pCD.

## Figures and Tables

**Table 1:** Comparisons of fistula MRI parameters (3 and 1.5 T MRI platforms) and clinical indicators before and after treatment. Data are presented as mean (SD). The volumes of the fistula defined on the MTR images before and after 12 weeks of treatment are also presented. The estimated mean differences mean (SEM) and 95% CI between the visits are also given.

	N	Baseline	12 Weeks	Estimated Difference of Mean	95% Confidence Interval
**MRI parameters on 3 T**					
MTR	18	0.32 (0.03)	0.32 (0.05)	0.002 (0.008)	−0.015, 0.019
ADC (×10^−3^ mm^2^/s)	17	1.48 (0.30)	1.46 (0.24)	−0.02 (0.05)	−0.14, 0.09
T2 (s)	18	0.082 (0.018)	0.077 (0.019)	−0.006 (0.004)	−0.013, 0.002
T1 (s)	17	2.24 (0.69)	1.98 (0.76)	−0.27 (0.12)	−0.530, −0.002
ETM *K*^trans^ (min^–1^)	17	0.10 (0.05)	0.11 (0.06)	0.008 (0.009)	−0.011, 0.027
ETM *v_e_*	17	0.30 (0.11)	0.34 (0.13)	0.043 (0.024)	−0.008, 0.094
ETM *v_P_*	17	0.0040 (0.0050)	0.0041 (0.0057)	0.0002 (0.0016)	−0.0032, 0.0035
Fistula Volume from MTR sequence (mL)	18	16.9 (22.0)	13.2 (20.9)	−3.7 (3.2)	−10.4, 3.1
**MRI parameters on 1.5 T**					
MTR	18	0.38 (0.08)	0.40 (0.08)	0.018 (0.023)	−0.029, 0.067
ADC (×10^−3^ mm^2^/s)	16	1.27 (0.29)	1.42 (0.29)	0.14 (0.06)	0.02, 0.27
Van Assche score	18	15 (6)	12 (4)	−3 (1)	−6, 0
Fistula volume from MTR sequence (mL)	18	14.9 (19.8)	14.4 (25.9)	−0.5 (2.8)	−6.3, 5.3
**Clinical indicators**					
PDAI	18	8 (3)	5 (3)	−3 (1)	−5, −2
CRP (mg/L)	18	17 (18)	6 (5)	−11 (4)	−20, −2

**Table 2:** Graphical representation of each individual patient data changes for all clinical and MRI parameters. Shading: decrease/increase is expected as a response to treatment—white. No change in parameter/score—grey. Decrease/increase is opposite to what is expected as response to treatment—black.

Patient No.	PDAI Change	CRP Change	VAS Change	3 T MTR Change	3 T ADC Change	3 T T2 Change	1.5 T MTR Change	1.5 T ADC Change	3 T Fistula Volume Change	1.5 T Fistula Volume Change
1	↓	↓	↑	↑↑	→	↓	↑	→	↑	↑↑
2	↓↓	↓↓	↓↓	↑↑	→	↓	↑	→	↓↓	↓↓
3	↑	→	→	↑	↓↓	↓↓	↓↓	→	→	→
5	↓↓	→	↑	↓	→	→	→	↑↑	→	↑↑
6	↓↓	↓	↓↓	↓↓	→	↑↑	↓	→	↓	↓↓
7	↓↓	↓	↑↑	→	→	↑	↑	↓	→	→
8	↓↓	↓↓	↓↓	↑	↓	↓↓	→	↑↑	↓↓	↓↓
9	↓↓	→	↓↓	→	→	→	↑	↑↑	↓↓	↓
11	↓↓	↓↓	↓↓	↑	→	↓↓	↑↑	↑↑	↓↓	↓↓
12	↓↓	↓↓	↓↓	↑	→	↓↓	→	→	↑↑	↑
13	↓↓	↓↓	↓	↓	↑↑	↓↓	→	↑↑	↓	→
14	↓↓	↓	↓↓	→	→	↓	↑↑	→	↓	↑
15	↓↓	↓	↓↓	→	↑	↓	→	→	↑↑	↑
16	↓↓	→	↓↓	→	↓	↓↓	↑	N/A	→	→
17	↓↓	↓↓	↓↓	→	↑	→	→	→	→	↓
18	↓↓	↓	↑	→	N/A	↑↑	↓↓	→	→	→
19	↑	↑	→	→	→	↓	→	N/A	↓	↓
20	↑	→	↑	→	→	→	→	→	↑	↑

PDAI—single arrow: change in score, double arrow: meaningful change in score defined as >0.5 SD Baseline; CRP—single arrow: change in score, double arrow: meaningful change in score > 0.5 SD Baseline; VAS—single arrow: change in score, double arrow: meaningful change in score >0.5 SD Baseline; All MRI scores—no change indicated if both values lie within the % LOA calculated from the muscle tissue, single arrow: change 1–2 × LOA, double arrow: change >2 × LOA; Volume—single arrow: change <50% of visit 1 data but larger than 1 mL absolute volume change, double arrow: change more than 50% of visit 1 and larger than 1 mL absolute volume change.

## Data Availability

Data are available on request from the corresponding author.

## Supplementary Materials

The following are available online at https://www.mdpi.com/article/10.3390/diagnostics11112135/s1, Figure S1: Consort Diagram for MAP study, Table S1: Participants’ demographic and clinical data for all subjects included in the MRI analysis, Figures S2–S4: Individual data plots before and 12 weeks after treatment, Table S2: MRI Parameters Pearson Correlation Coefficient r.

## Appendix A

MRI sequence information.

**Table A1:** MRI sequence parameter information for 1.5 T GE scanner (Nottingham site).

Parameters	Imaging Plane	TR/TE ms	FOV mm^2^	Slice Thickness (Gap) mm	Matrix Acquired	Matrix Recon
T2w FSE	Sagittal	4500/93	260 × 260	4 (0.4)	288 × 244	512 × 512
T2w FSE	Coronal	7000/114	320 × 320	4 (0.4)	320 × 224	512 × 512
T2w FSE	Axial	9740/114	320 × 320	4 (0.4)	320 × 224	512 × 512
STIR FSE	Coronal Oblique	4100/57	270 × 270	3 (0.3)	256 × 224	256 × 256
STIR FSE	Axial Oblique	3900/58	270 × 270	3 (0.3)	256 × 224	256 × 256
MTR GRE	Coronal Oblique	33/3.8	380 × 380	4 (0.4)	288 × 244	512 × 512
DWI	Axial	2000/71	350 × 350	4 (0.4)	144 × 144	256 × 256

T2w—T2 weighted, FSE—fast spin echo, STIR—short tau inversion recovery, MTR—magnetisation transfer ratio, GRE—gradient recalled echo, DWI—diffusion weighted imaging, TR—repetition time, TE—echo time, FOV—filed of view, Gap—spacing between slices.

**Table A2:** MRI sequence parameter information for 1.5 T Philips scanner (London and Manchester sites).

Parameters	Imaging Plane	TR/TE ms	FOV mm^2^	Slice Thickness (Gap) mm	Matrix Acquired	Matrix Recon
T2w FSE	Sagittal	3163/90	260 × 260	4 (0.4)	324 × 255	384 × 384
T2w FSE	Coronal	4291/109	320 × 320	4 (0.4)	320 × 240	400 × 400
T2w FSE	Axial	4517/106	320 × 320	4 (0.4)	320 × 257	400 × 400
STIR FSE	Coronal Oblique	4643/60	270 × 270	3 (0.3)	300 × 240	400 × 400
STIR FSE	Axial Oblique	4916/60	270 × 270	3 (0.3)	300 × 240	384 × 384
MTR GRE	Coronal Oblique	47/4.2	300 × 300	4 (0)	272 × 231	336 × 336
DWI	Axial	2500/64	350 × 350	4 (0.4)	144 × 144	160 × 160

**Table A3:** MRI sequence parameter information for 3.0 T Philips scanners (Nottingham and London Sites).

Parameters	Imaging Plane	TR/TE ms	FOV mm^2^	Slice Thickness (Gap) mm	Matrix Acquired	Matrix Recon
T2w FSE	Sagittal	5417/100	260 × 260	4 (0.4)	228 × 220	320 × 320
T2w FSE	Coronal	6500/100	320 × 320	4 (0.4)	280 × 252	384 × 384
T2w FSE	Axial	10,472/100	320 × 320	4 (0.4)	280 × 220	384 × 384
T2w FSE SPAIR	Coronal Oblique	4526/80	270 × 270	3 (0.3)	272 × 237	288 × 288
T2w FSE SPAIR short echo time	Coronal Oblique	4526/7.3	270 × 270	3 (0.3)	272 × 237	288 × 288
T2w FSE SPAIR	Axial Oblique	4385/80	270 × 270	3 (0.3)	244 × 244	352 × 352
DWI	Axial	6154/88	320 × 296	4 (0.4)	160 × 146	192 × 192
MTR 3D GRE	Coronal Oblique	72/4.5	298 × 298	3 (0)	248 × 228	384 × 384
3D T1w GRE VFA (2°,10°,20°,30°)	Coronal Oblique	9.2/1.8	280 × 280	4 (0)	216 × 216	288 × 288
T1w GRE Pre/Post-contrast FATSAT	Coronal Oblique	3.2/1.5	260 × 260	1.6 (0)	162 × 162	288 × 288
T1w GRE Pre/Post contrast FATSAT	Axial Oblique	3.2/1.5	260 × 260	1.6 (0)	162 × 162	288 × 288
DCE-4D THRIVE with Fat Sat	Coronal Oblique	3.3/1.6	280 × 280	4 (0)	216 × 216	288 × 288

SPAIR—spectral attenuated inversion recovery, FATSAT—fat saturation, T1w—T1 weighted, DCE—dynamic contrast enhancement, THRIVE—T1-weighted high-resolution isotropic volume examination.

**Table A4:** MRI sequence parameter information for 3.0 T Philips scanner (Manchester Site).

Parameters	Imaging Plane	TR/TE ms	FOV mm^2^	Slice Thickness (Gap) mm	Matrix Acquired	Matrix Recon
T2w FSE	Sagittal	8978/100	260 × 260	4 (0.4)	228 × 220	320 ×320
T2w FSE	Coronal	10,774/100	320 × 320	4 (0.4)	280 × 252	384 × 384
T2w FSE	Axial	17,359/100	320 × 320	4 (0.4)	280 × 220	384 × 384
T2w FSE SPAIR	Coronal Oblique	10,631/80	270 × 270	3 (0.3)	272 × 237	288 × 288
T2w FSE SPAIR short echo time	Coronal Oblique	10,631/7.3	270 × 270	3 (0.3)	272 × 237	288 × 288
T2w FSE SPAIR	Axial Oblique	4627/80	270 × 270	3 (0.3)	244 × 244	352 × 352
DWI	Axial	4547/77	320 × 296	4 (0.4)	160 × 146	192 × 192
MTR 3D GRE	Coronal Oblique	144/4.5	298 × 298	3 (0)	248 × 228	384 × 384
3D T1w GRE VFA (2°,10°,20°,30°)	Coronal Oblique	9.2/1.8	280 × 280	4 (0)	216 × 216	288 × 288
T1w GRE Pre/Post-contrast FATSAT	Coronal Oblique	3/1.4	260 × 260	1.6 (0)	162 × 162	288 × 288
T1w GRE Pre/Post contrast FATSAT	Axial Oblique	3/1.4	260 × 260	1.6 (0)	162 × 162	288 × 288
DCE-4D THRIVE with Fat Sat	Coronal Oblique	3.3/1.6	280 × 280	4 (0)	216 × 216	288 × 288

Manchester sequences had different TR/TE values for some of the scans compared to the other 2 sites due to the lack of multi-transmit capability of the scanner.

## Appendix B

Methodology to obtain fat fraction and water T1 from Variable FA GRE scan and fat-suppressed DCE sequences.

DCE-MRI is commonly acquired using a spoiled gradient-echo (SPGR) dynamic acquisition, preceded by variable flip angle (VFA) spoiled gradient echo acquisition designed to generate a pre-contrast T_1_ map according to the steady state SPGR equation:

(A1) $$S_{v}=M_{0}\sin A\left(1-e\times p\left(-TR/T_{1}{}_{v}\right)\right)/\left(1-\cos Ae\times p\left(-TR/T_{1}{}_{v}\right)\right)$$

where *S_v_* describes the total voxel signal, *T*_1*v*_ is the product of voxel proton density and gain, *A* is the flip angle and *T*_1*v*_ is the voxel longitudinal relaxation time. Given known repetition time *TR*, a set of known *A*, and measured *S_v_*, *T*_1*v*_ and *M*_0_ can be estimated using a nonlinear fit or appropriate linearisation of Equation (A1).

We consider the case where there are two subvoxel components of fat and water and describe them using Equation (A2), whereby

(A2) $$S_{v}=M_{0}\sin A\left[\left(1-F\right)\left(1-e\times p\left(-TR/T_{1}{}_{w}\right)\right)/\left(1-\cos Ae\times p\left(-TR/T_{1}{}_{w}\right)\right)\;\;\;\;\;\;\;\;+F\left(1-e\times p\left(-TR/T_{1}{}_{f}\right)\right)/\left(1-\cos Ae\times p\left(-TR/T_{1}{}_{f}\right)\right)\right]$$

where *T*_1*w*_ is the *T*_1_ of the water component, *T*_1*f*_ is the *T*_1_ of the fat component and *F* is the fat fraction.

Where the precontrast VFA images are acquired using no fat saturation, but the dynamic time series is acquired using fat saturation and flip angle *A*(*d*), we apply the following approach to calculate a fat fraction map and perform a subsequent DCE-MRI analysis:

1. Calculate initial estimates for *T*_1*v*_ and *M*_0_ using a linearisation of Equation (A1).
2. Calculate the predicted non-fat-supressed signal *S_v_*(*A*(*d*)), using the results of step 1 and Equation (A1).
3. From the mean signal of the pre-contrast portion of the fat-supressed dynamic time series images *S_d_* and the predicted non-fat-supressed signal *S_v_*(*A*(*d*)), calculate an initial estimate of the fat fraction *F* as
  (A3) $$F=1-S_{d}/S_{v}\left(A\left(d\right)\right)$$
4. Calculate an initial estimate for *T*_1*w*_ using the relationship
  (A4) $$S_{w}=M_{0}\left(1-F\right)\sin A\left(1-e\times p\left(-TR/T_{1}{}_{w}\right)\right)/\left(1-\cos Ae\times p\left(-TR/T_{1}{}_{w}\right)\right)$$
5. Input these initial estimates to a nonlinear fit of Equation (A2) to the VFA images using an assumed *T*_1*f*_ = 0.3 s to find *M*_0_, *T*_1*w*_ and *F*.

The *T*_1*w*_ measurements can be used as baseline *T*_1_ measurements for calculation of the dynamic *T*_1_ measurements and subsequent tracer kinetic modelling, whilst *F* provides the fat fraction map.
