# Supplementary material for: Quantitative Magnetic Resonance Imaging in Perianal Crohn’s Disease at 1.5 and 3.0 T: A Feasibility Study
Source: Diagnostics (Basel). 2021 Nov 17;11(11):2135. doi: 10.3390/diagnostics11112135 (PMC8624877; doi:10.3390/diagnostics11112135)
Supplement: Supplementary file 1 [file diagnostics-11-02135-s001.zip › diagnostics-1447127-supplementary.pdf]

Supplementary Materials

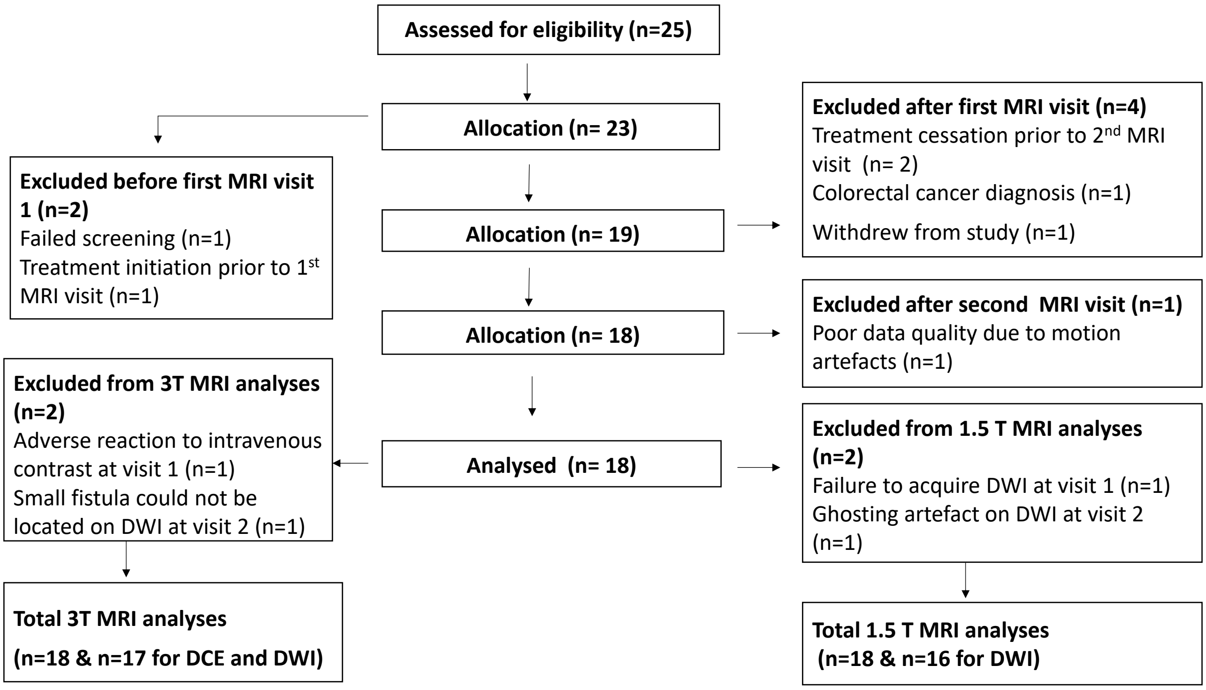

Figure S1. Consort Diagram for MAP study.

Table S1. Participants demographic and clinical data for all subjects included in the MRI analysis.

**Patient No.**	**Age (Years)**	**Sex**	**PDAI Visit 1**	**PDAI Visit 2**	**CRP Visit 1**	**CRP Visit 2**	**VAS Visit 1**	**VAS Visit 2**	**Rectal Involvement**	**Fistula Complexity**	**Disease Location**	**H/O Surgery**	**Stoma**	**RV Fistula**
1	27	M	5	4	10	5	9	11	No	Complex	2	No	No	No
2	24	M	8	0	47	5	22	7	Yes	Complex	2	Yes	No	No
3	40	F	6	7	5	5	7	7	No	Simple	3	No	No	No
5	37	F	7	4	5	5	7	9	No	Simple	2	No	No	No
6	45	F	8	2	12	5	22	11	Yes	Complex	2	No	No	No
7	38	F	5	3	6	5	8	13	No	Simple	3	No	No	No
8	23	M	6	5	27	5	20	14	Yes	Complex	1	No	No	No
9	27	M	11	8	5	5	15	7	Yes	Simple	2	No	No	No
11	52	F	11	1	74	5	14	7	Yes	Simple	3	No	No	No
12	64	F	14	10	22	5	16	13	Yes	Complex	3	Yes	Yes	No

13	49	M	11	4	20	5	13	12	No	Complex	3	Yes	No	No
14	53	F	14	9	15	8	22	14	Yes	Complex	3	Yes	Yes	Yes
15	33	F	8	4	8	5	20	11	Yes	Complex	2	No	No	No
16	39	F	7	3	5	5	16	12	Yes	Simple	1	Yes	No	Yes
17	52	F	8	6	26	5	13	10	No	Complex	2	No	No	No
18	18	F	6	2	2	1	7	18	No	Simple	3	No	No	No
19	39	F	9	10	20	26	22	22	Yes	Complex	3	No	No	No
20	29	F	8	9	1	1	17	19	no	Simple	3	Yes	No	No

VAS: Van Assche Score; RV: Recto-vaginal.

Individual Data Plots before and 12 weeks after treatment.

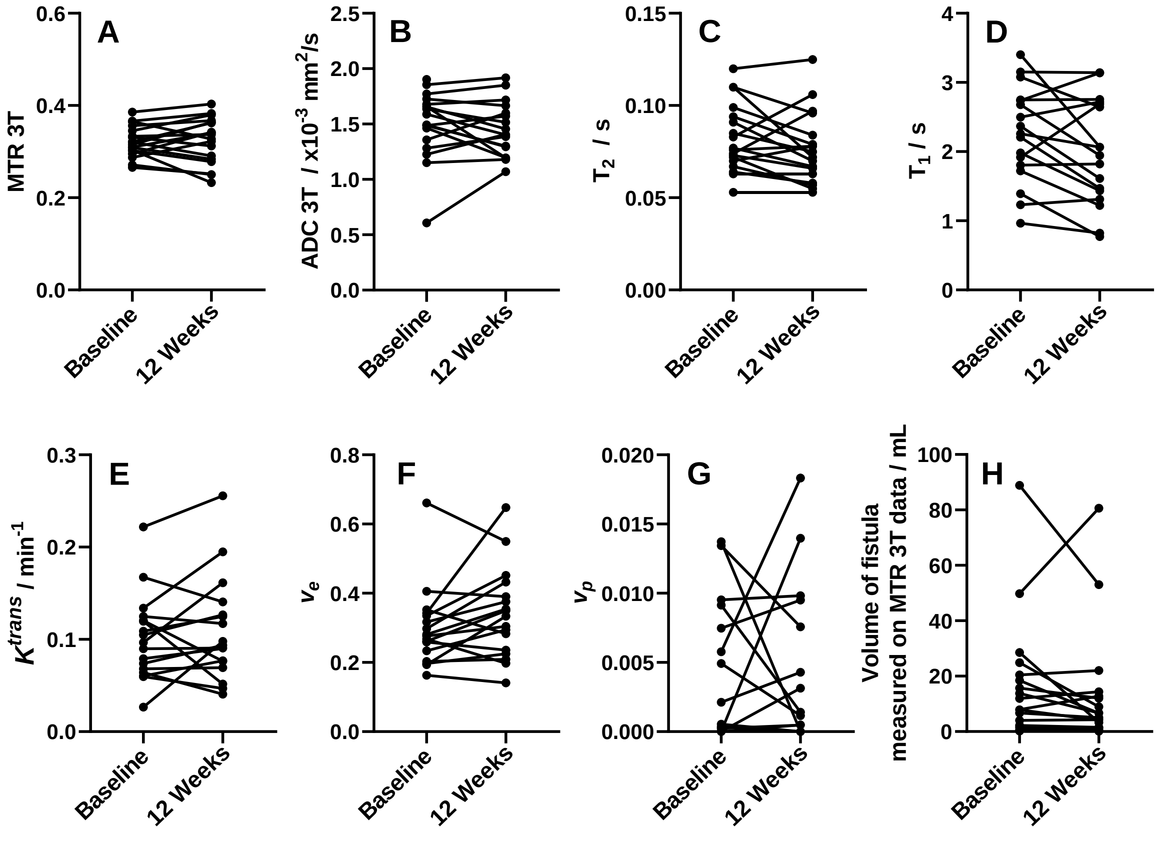

Figure S2. Individual data plots for 3T parameters measured before (Baseline) and after 12 weeks of treatment in the diseased tissue. (**A**) MTR, (**B**) ADC, (**C**) T2, (**D**) T1, (**E**) K^{trans}, (**F**) v_e, (**G**) v_p (**H**) Fistula volume measured from MTR data.

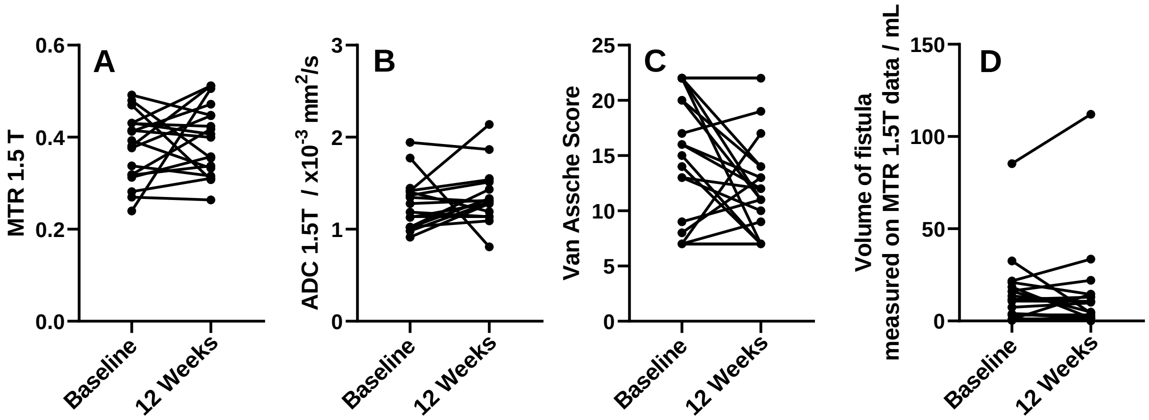

Figure S3. Individual data plots for 1.5T parameters measured before (Baseline) and after 12 weeks of treatment in the diseased tissue. (**A**) MTR, (**B**) ADC, (**C**) Van Assche Score, (**D**) Fistula volume measured from MTR data.

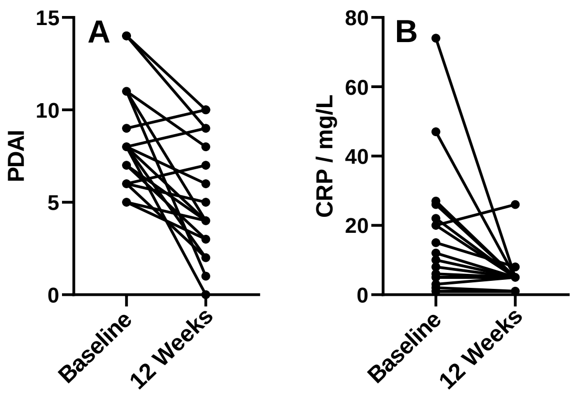

Figure S4. Individual data plots for clinical parameters measured before (Baseline) and after 12 weeks of treatment. (**A**) PDAI score, (**B**) CRP.

Table S2. MRI Parameters Pearson Correlation Coefficient r.

3T MRI Parameter Correlation							
	**ADC 3T ♦**	**T_2**	**T_1 ***	***K^{trans}* ***	***v_e* ***	***v_p* ***	**VAS**
MTR 3T	-0.51<br>−0.72 to -0.21	-0.59<br>−0.77 to −0.33	0.09<br>−0.25 to 0.42	-0.66<br>−0.82 to 0.42	-0.34<br>−0.61 to 0.00	-0.52<br>−0.73 to -0.22	-0.30<br>−0.57 to 0.03
ADC 3T ♦		0.35<br>0.02 to 0.61	0.32<br>−0.02 to 0.60	0.17<br>−0.19 to 0.48	-0.09<br>−0.42 to 0.27	0.11<br>−0.24 to 0.44	0.22<br>−0.12 to 0.52
T_2			0.53<br>0.23 to 0.73	0.16<br>−0.19 to 0.47	-0.19<br>−0.50 to 0.16	0.19<br>−0.16 to 0.50	0.58<br>0.32 to 0.77
T_1*				N/A	N/A	N/A	0.33<br>−0.01 to 0.60
*K^{trans}**					N/A	N/A	0.11<br>−0.24 to 0.43
v_e *						N/A	-0.14<br>−0.46 to 0.21

v_p *		0.23<br>−0.12 to 0.52

1.5 T MRI Parameter Correlation

	ADC 1.5T*	VAS
MTR 1.5T	−0.11<br>−0.44 to 0.23	−0.55<br>−0.74 to −0.27
ADC 1.5T *		0.00<br>−0.33 to 0.34

♦ n = 35, * n = 34, *♦combined n = 33, for the rest of the data n=36. 95% confidence intervals for the Pearson Correlation coefficient r, are shown in light grey shading. As K^{trans}, v_e and v_p were all derived from the same dataset which included the T_1 data, these parameters were not assessed for cross-correlation.
